# Physical Properties of Extrudates with Fibrous Structures Made of Faba Bean Protein Ingredients Using High Moisture Extrusion

**DOI:** 10.3390/foods11091280

**Published:** 2022-04-28

**Authors:** Katja Kantanen, Anni Oksanen, Minnamari Edelmann, Heikki Suhonen, Tuula Sontag-Strohm, Vieno Piironen, Jose Martin Ramos Diaz, Kirsi Jouppila

**Affiliations:** 1 Department of Food and Nutrition, University of Helsinki, Agnes Sjöbergin katu 2, FI-00014 Helsinki, Finland; anni.oksanen@helsinki.fi (A.O.); minnamari.edelmann@helsinki.fi (M.E.); tuula.sontag-strohm@helsinki.fi (T.S.-S.); vieno.piironen@helsinki.fi (V.P.); jose.ramosdiaz@helsinki.fi (J.M.R.D.); kirsi.jouppila@helsinki.fi (K.J.); 2 Department of Physics, University of Helsinki, Gustaf Hällströmin katu 2, FI-00014 Helsinki, Finland; heikki.suhonen@helsinki.fi

**Keywords:** faba bean protein isolate, faba bean protein concentrate, high moisture extrusion, fibrous protein structure, meat analogue

## Abstract

Faba bean is a potential ingredient due to its high protein yield and its possible cultivation in colder climate regions. In this study, meat analogues made from faba bean protein isolate (FPI) and concentrate (FPC) blends were produced using high moisture extrusion. The aim of this study was to investigate the effect of the FPI content (FPIc), feed water content (FWC), and temperature of the long cooling die (LT) during extrusion on the mechanical and physicochemical properties as well as on the structure of the meat analogues. Increased FPIc resulted in higher values in hardness, gumminess, chewiness, and cutting strengths as well as in darker colour and decreased water absorption capacity. The effect of increased FWC on these properties was weaker and the opposite. Images from microtomography revealed that higher FPIc led to a less organised fibrous structure. In conclusion, fibrous structures can be achieved by utilising a mixture of faba bean protein ingredients, and a higher FPC content seemed to promote fibre formation in the meat analogue.

## 1. Introduction

The current need for a shift towards a plant-based diet has created new ways of utilising plant proteins, leading to the development of new plant-based meat alternatives [1]. The most common raw materials used in these types of products are soy protein, wheat gluten, and pea protein [2]. However, consumer interest in soybean as a protein source has decreased in recent years [3]. Faba bean could offer a domestically grown alternative to imported soybean due to its high protein yield and ability to grow in colder climates, particularly in boreal regions such as Finland [4]. The cultivation of faba bean would also benefit crop rotation by decreasing the need for nitrogen fertiliser and by preventing cereal disease cycles [5].

Faba bean (*Vicia faba*) contains 19–39% protein, depending on the genotype, and around 80% of its storage proteins are globulins of the 7S and 11S types [6]. Faba bean protein ingredients have functionalities similar to those of soybean protein, including water and oil holding capacity, foam stability, whippability, and gelation; therefore, they could be a good source of plant protein for various food applications [7,8,9]. Faba bean is still underutilised in food products, although promising applications involving yoghurt, tofu-like products, and meat analogues have been investigated recently [10,11,12,13,14]. Meat analogues are defined as products that can be used as part of a meal in similar ways as meat, and they possess functionalities, properties, and sensory qualities comparable to meat [15]. Meat resembling fibrous plant protein structures can be produced by texturisation using high moisture extrusion technology [16]. During high moisture extrusion, shear forces and pressure at high temperatures lead to the unfolding of proteins, which then begin to cross-link and form new protein–protein interactions in the long cooling die due to the lowered temperature. In the long cooling die, the temperature gradient and flow behaviour of the melt along with solidification contribute to the formation of fibrous meat-mimicking structures.

Plant protein ingredients, such as protein concentrates and isolates, are used in the production of meat analogues [17]. Protein isolates are produced with wet fractionation and contain around 75–90% protein, and protein concentrates are produced with dry fractionation and contain around 48–65% protein. Due to their different production methods, protein isolates and concentrates can possess different techno-functional properties [18,19]. According to Vogelsang-O’Dwyer et al. [19], the dry fractionated faba bean protein concentrate (FPC) had better gelling and foaming abilities than the faba bean protein isolate (FPI). They speculated that this could be due to the better protein solubility of the FPC. Even though the FPC had superior functionalities, they noted that it also contained a higher amount of antinutrients, which were almost completely absent in the FPI. Moreover, the differences in the functionalities of faba bean protein ingredients can be the result of different protein extraction methods, genotypes, and environmental conditions [20,21].

To the best of our knowledge, only a few studies have assessed the high moisture extrusion of faba bean protein ingredients. Saldanha do Carmo et al. [12] studied the high moisture extrusion of dry fractionated FPC, and Ferawati et al. [13] examined the differences between FPI and FPC in high moisture extrusion. Kim et al. [10] compared the cooling and rehydration methods of faba bean protein-containing meat analogues produced with high moisture extrusion. Kim et al. [14] studied the structuring of burger patties from faba bean protein containing meat analogues produced with high moisture extrusion. Given the potential of faba bean as a protein source, and to better understand faba bean protein ingredients in the texturisation process and in the structure formation of meat analogues, our objective was to study the high moisture extrusion of a mixture of FPI and FPC. The focus was to study the effects of FPI content of solids, feed water content, and temperature of the long cooling die in high moisture extrusion on the mechanical properties, structure, and physicochemical properties of meat analogues.

## 2. Materials and Methods

### 2.1. Materials

FPI and FPC were used as raw materials. The chemical composition, water solubility index (WSI), water absorption index (WAI), and particle sizes of the ingredients used are presented in Table 1. FPI (Faba Bean Protein 90-C-EU) was obtained from AGT Food ingredients, Inc. (Regina, Canada). Dry fractionated FPC (Fava Bean Flour P60) was obtained from Suomen Viljava Ltd. (Helsinki, Finland). For the oil absorption capacity analysis, rapeseed oil (Keiju; Bunge Finland Ltd., Raisio, Finland) was purchased from local store.

The analysis of protein content followed the Kjeldahl method using Kjeltec digestor and analyser units (FOSS analytical A7S, Hilleröd, Denmark). The sample (0.1 g, three replicates) was digested in sulphuric acid (18 mL, at 400 °C) for 1.5 h. After cooling and water addition, the hydrolysed sample was analysed for nitrogen content, and a nitrogen-to-protein conversion factor of 5.4 was used to calculate the protein content. Fat content was expressed as the sum of analysed fatty acid methyl esters. Fat was extracted from a 1 g sample with ethanol in triplicate using accelerated solvent extraction (Dionex ASE-200, Dionex Corporation; Sunnyvale, CA, USA) [22]. Extracts were evaporated to dryness and redissolved in heptane. Fatty acids were methylated and quantified as their methyl esters with the gas chromatographic method according to Liu, Lampi, and Ertbjerg [23]. The soluble and insoluble dietary fibre content was determined following the AOAC 991.43 method [24], with one exception. In the enzymatic digestion of protein, 200 µL of protease solution (50 mg/mL; P3910 Sigma-Aldrich (St. Louis, MO, USA)) instead of 100 µL was used. The WAI and WSI of the ingredients were measured according to the method reported by Dansby and Bovell-Benjamin [25]; deionised water was used as the solvent. The particle diameter of the raw materials (flour) was measured with a laser diffraction particle size analyser (Mastersizer 3000 SM, Malvern Instruments Ltd., Worcestershire, UK) connected to a dry powder dispersion unit (Aero S); the refractive index was set at 1.46.

### 2.2. High Moisture Extrusion

Three mixtures with different FPI:FPC ratios were extruded at varying amounts of feed water and temperatures of the long cooling die, according to the split-plot Box–Behnken experimental design (Table 2). Extrusion was conducted using a twin-screw extruder (Rheomex PTW24/28p, Polylab System, Rheocord 300p; Thermo Haake, Karlsruhe, Germany). The screw length was 672 mm, screw diameter was 24 mm, and the length-to-diameter ratio was 28:1. More detailed measurements can be found in the study by Ramos Diaz et al. [26]. The screw speed was set to 400 rpm. The barrel of the extruder was divided into seven zones, six of which were temperature-controlled. Temperature profiles of zones 1–6 were adjusted to 25, 40, 80, 100, 120, and 150 °C, respectively. The seventh zone (TS-D1) had a temperature of 150 °C. A long cooling die (flat cooling nozzle FKD75, DIL Deutsches Institut für Lebensmitteltechnik, Quakenbrück, Germany) was attached to the seventh zone, and the inner dimensions were 50 mm × 15 mm × 800 mm (width × height × length). The temperature of the long cooling die ranged from 40 to 80 °C (Table 2). Reverse osmosis water was used as the liquid, and the total mass feed rate was 50 g/min.

Pressure and torque were recorded during extrusion, and the samples were collected when the torque was levelled off under the conditions of each experiment. The samples were cut into 20 cm pieces upon exit from the long cooling die, placed in polyethylene zip-lock bags, cooled at room temperature, and stored in a freezer at −20 °C. Before further analyses, the samples were thawed at room temperature overnight.

### 2.3. Mechanical Properties

#### 2.3.1. Texture Profile Analysis

Texture profile analysis (TPA) was conducted using a Texture Analyser (Stable Micro Systems, Godalming, Surrey, England). The samples for the TPA were cut into cubes, with dimensions of 24 mm (width) × 24 mm (length) × 14 mm (height), and five replicates from each sample were measured. Samples were placed on a flat platform sample holder, and a cylindrical probe (diameter, 36 mm) was used to compress the sample twice using a 50 kg load cell. The settings for the compressions were: pre-test speed, 1 mm/s; test speed, 1 mm/s; post-test speed, 5 mm/s; deformation distance, 7 mm (50% of the height of the sample); resting time, 5 s; trigger force, 5 g. Hardness, gumminess, springiness, and chewiness were determined from the force–distance curve using Equations (1)–(4).

(1) $$\mathrm{Hardness}\;\left[N\right]=\mathrm{maximum}\;\mathrm{force}\;\mathrm{of}\;\mathrm{the}\;\mathrm{first}\;\mathrm{bite}$$

(2) $$\mathrm{Gumminess}\;\left[N\right]=\frac{\mathrm{area}\;\mathrm{under}\;\mathrm{the}\;\mathrm{deformation}\;\mathrm{curve}\;\mathrm{of}\;\mathrm{the}\;\sec\mathrm{ond}\;\mathrm{bite}}{\mathrm{area}\;\mathrm{under}\;\mathrm{the}\;\mathrm{deformation}\;\mathrm{curve}\;\mathrm{of}\;\mathrm{the}\;\mathrm{first}\;\mathrm{bite}}\times \mathrm{hardness}$$

(3) $$\mathrm{Springiness}=\frac{\mathrm{distance}\;\mathrm{of}\;\mathrm{the}\;\mathrm{detected}\;\mathrm{height}\;\mathrm{during}\;\mathrm{the}\;\sec\mathrm{ond}\;\mathrm{bite}}{\mathrm{distance}\;\mathrm{of}\;\mathrm{the}\;\mathrm{detected}\;\mathrm{height}\;\mathrm{during}\;\mathrm{the}\;\mathrm{first}\;\mathrm{bite}}$$

(4) $$\mathrm{Chewiness}\;\left[J\right]=\mathrm{gumminess}\times \mathrm{springiness}$$

#### 2.3.2. Cutting Strength

The cutting strength was analysed using a Texture Analyser (Stable Micro Systems, Godalming, Surrey, UK). The cutting strength was measured from directions both perpendicular and longitudinal to the flow of the material inside the long cooling die. The dimensions of the samples were 20 mm (width) × 30 mm (length) × 10 mm (height), and five replicates from each sample were measured. A one-sided razor blade (width: 39 mm, height: 19 mm) was used to cut across the sample, either horizontally in the direction of the flow (longitudinal) or vertically in the direction of the flow (perpendicular). The settings for the measurements were: load cell, 5 kg; pre-test speed, 1 mm/s; test speed, 1 mm/s; post-test speed, 5 mm/s; cutting distance, 10 mm. The cutting strength was determined as the maximum force from the force-distance curve. Anisotropy index (AI) was calculated as the ratio of the perpendicular-to-longitudinal cutting forces.

### 2.4. Colour

The colour of the samples was measured using a Minolta Chroma Meter CR-400 (Konica Minolta Sensing, Inc., Osaka, Japan). The colour was expressed as CIE-lab parameters L*, a*, and b*. Parameter L* expresses the lightness of the sample (0 = black and 100 = white). Negative values for parameter a* express the greenness, and positive values express the redness of the sample. Negative values for parameter b* express the blueness, and positive values express the yellowness of the sample. Ten replicates of each sample were measured.

### 2.5. Water Absorption Capacity (WAC) and Oil Absorption Capacity (OAC)

Analyses of water and oil absorption capacities were conducted according to the method by Lin et al. [27], with some modifications. Samples were cut into pieces with dimensions of 20 mm (width) × 30 mm (length) × 10 mm (height), and three replicates were measured from each sample. The samples were dried in an airflow convection oven (Termaks, Bergen, Norway) for 24 h at 40 °C. After drying, the samples were rehydrated with either 40 mL of deionised water or rapeseed oil in a 50 mL falcon tube and placed in a water bath at 50 °C for 16 h. The water and oil absorption capacities were calculated using Equation (5).

(5) $$\mathrm{WAC}\;\mathrm{or}\;\mathrm{OAC}\;\left(\%\right)=\left(\frac{\mathrm{weight}\;\mathrm{after}\;\mathrm{rehydration}-\mathrm{weight}\;\mathrm{before}\;\mathrm{rehydration}\;}{\mathrm{weight}\;\mathrm{before}\;\mathrm{rehydration}}\right)\times 100$$

### 2.6. Water Hydration Capacity (WHC)

For the analysis of the WHC, the samples were first dried and milled. For drying, the samples were shredded into small pieces and then dried in an airflow convection oven (Termaks, Bergen, Norway) for 18 h at 50 °C. The samples were then placed in a vacuum desiccator (desiccant: phosphorus pentoxide, P_2_O_5_; Merck, KGaA, Darmstadt, Germany) to cool for 2 h. After cooling, the samples were milled using an ultra-centrifugal mill (Retsch ZM 200, Haan, Germany) at 10,000 rpm with a 0.5 mm sieve. The WHC was analysed according to the AACC Method 56-30. For the measurement of approximate water hydration capacity, 5 g of the sample was weighed into a 50 mL falcon tube, and deionised water was added until the sample was wetted. The samples were then centrifuged at 2000× *g* for 10 min at 6 °C. The approximate WHC was calculated using Equation (6). For the analysis of the actual WHC, the sample was weighed into four falcon tubes. The weight of the sample was determined using Equation (7). Deionised water was added to the four falcon tubes at volumes 1.5 and 0.5 higher as well as 1.5 and 0.5 lower than the average volume of water calculated according to Equation (8). The tubes were then vortexed and stirred with a glass rod for 2 min.

(6) $$\mathrm{Approximate}\;\mathrm{WHC},\frac{\mathrm{mL}}{g}=\frac{\left(\mathrm{weight}\;\mathrm{of}\;\mathrm{tube}+\mathrm{sediment}\;\right)-\left(\mathrm{weight}\;\mathrm{of}\;\mathrm{tube}+5.0\right)}{5}$$

(7) $$\mathrm{Weight}\;\mathrm{of}\;\mathrm{material}=\frac{15}{\mathrm{approximate}\;\mathrm{WHC}+1}$$

(8) Average volume of water=15−weight of material

The samples were then centrifuged at 2000× *g* for 10 min at 6 °C. Two adjacent tubes, one with supernatant and one without, were used to calculate the WHC. The WHC was determined as the average of the water volumes added to these two adjacent tubes divided by the weight of the material in the tube. For the analysis of WHC, three replicates from each sample were measured.

### 2.7. Stereomicroscopy

Before imaging, the samples were cut into 6 cm-long pieces. A small cut was made along the side of the piece, and then the piece was split into two to reveal the inner structure. Pictures of the samples were taken with a digital camera DSLR body (Nikon 7200, Tokyo, Japan) attached to a telephoto lens (18–400 mm, f/3.5–6.3, DI II VC HLD zoom objective, Tamron Co., Ltd., Saitama, Japan) using the following settings: distance to the object, 20 cm; magnification, 18 mm; aperture, 7.1; shutter speed, 250; ISO, 1000. Pictures were taken in a light-controlled cabinet using warm day light (D50). Stereomicroscopy imaging was performed on the same sample pieces using a Stemi DV4 stereomicroscope (Carl Zeiss MicroImaging, Göttingen, Germany), enlarging them 10 times with the digital zoom. The pictures were processed using the Zeiss Zen 3.2 (blue edition) software (Carl Zeiss Microscopy GmbH, München, Germany).

### 2.8. Microtomography

Before microtomography imaging, the samples were cut into pieces with dimensions of 20 mm (width) × 30 mm (length) × 10 mm (height). The cut pieces were then placed in a freezer at −70 °C for 24 h and then freeze dried for 3 days using a Lyovac GT 2 freeze-dryer (Amsco Finn-Aqua GmbH, Hürth, Germany) at a pressure below 0.5 mbar. The freeze-dried samples were imaged with an X-ray micro-CT system phoenix nanotom|s (phoenix|X-ray Systems + Services GmbH, currently part of Waygate Technologies, owned by Baker Hughes, Huerth, Germany). The samples were placed in a small plastic cup and supported by Styrofoam balls to keep them still during the scan. The X-ray generator voltage was 60 kV, the current was 150 μA, and no filter was used. For each sample, 1600 projection images were taken during a 360° rotation, with a 3 × 500 ms exposure time for each projection (total imaging time was 53 min per sample). The data were reconstructed into 3D volumes using the phoenix datos|x 2 reconstruction software version 2.4.0 (phoenix|X-ray Systems + Services GmbH). The final voxel size in the images was 20 µm. The images were analysed on Fiji/ImageJ [28,29]. First, random noise was reduced by non-local means denoising [30,31], and then the void portion was identified by choosing a threshold value (the same for all samples), below which the grey scale values were interpreted as the void space. The thickness distribution for the void space was calculated using the ImageJ LocalThickness plugin [32]. The frequency distributions for these discrete thickness distributions were then calculated, and these were converted to probability distribution by normalizing the sum to 1.

### 2.9. Statistical Analyses

Average values from the results of the mechanical and physicochemical properties were used in the statistical analyses. Multiple linear regression analysis (MLR) was used to investigate the linear and quadratic effects as well as the interaction terms of the independent variables (FPIc, LT, and FWC) on the response variables of the mechanical and physicochemical properties of the meat analogues. Partial least squares regression analysis (PLSR) was used to investigate the relationships between independent variables and response variables using the first two components of the model. The MLR and PLSR models were calculated using MODDE 12.1 and SIMCA 15.0 (Sartorius Corporate Administration, Göttingen, Germany), respectively.

## 3. Results and Discussion

### 3.1. Regression Models

According to the MLR models, FPIc had a statistically significant effect on almost all response variables and FWC content on many response variables, but LT influenced only a few response variables. The coefficients of determination (R^2^) of the MLR models were high, except for OAC and WHC (Table 3). The coefficient of prediction (Q^2^) was very low for pressure, springiness, OAC, WHC, a*, and b*. For springiness, OAC, and WHC, the regression was not statistically significant, and for hardness, gumminess, chewiness, and a*, the lack of fit was statistically significant. The MLR models indicated that FPIc had a positive effect on all the other response variables except L*, on which the effect was negative. The effect of FWC was the opposite, negatively affecting all other response variables except L*. Additionally, LT showed a slightly positive effect on WAC.

**Figure 1:**
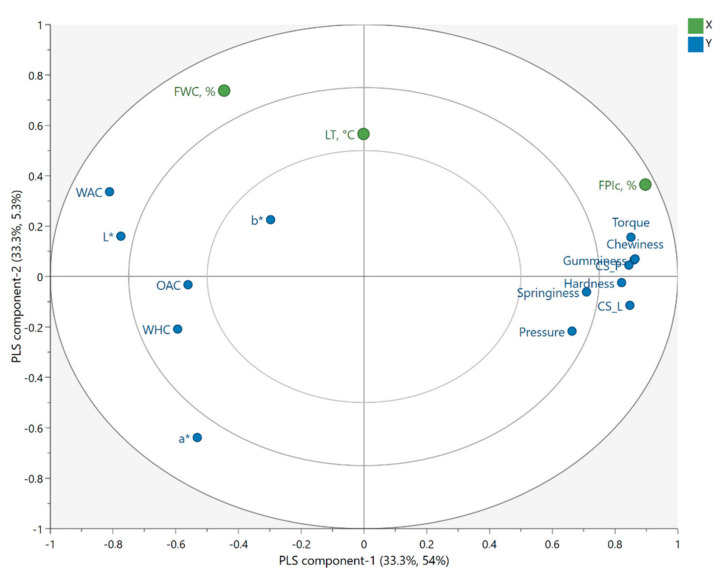
PLS weight plot showing the effects of independent variables of faba bean protein isolate content (FPIc), feed water content (FWC), and long cooling die temperature (LT) on the response variables of torque, pressure, hardness, chewiness, gumminess, springiness, cutting strength longitudinal (CS_L), cutting strength perpendicular (CS_P), water absorption capacity (WAC), oil absorption capacity (OAC), water hydration capacity (WHC), lightness (L*), redness (a*), and yellowness (b*). PLS component-1 explained 33.3% and 54% of the variation in x and y, respectively, and PLS component-2 explained 33.3% and 5.3% of the variation in x and y, respectively.

The PLSR model included the first two components, and R^2^ and Q^2^ for the model were 0.593 and 0.483, respectively. The model provided a statistically significant explanation for the variations in the response variables of torque, hardness, gumminess, chewiness, cutting strengths, WAC, and L* (Appendix A, Table A1). The variable importance in projection (VIP) values of the independent variables were the highest for FPIc (1.49), the second highest for FWC (0.83), and the lowest for LT (0.29), indicating that FPIc was the most important independent variable affecting the response variables. The PLS weight plot (Figure 1) shows that the mechanical properties, torque, and pressure positively correlated with each other and were positively affected by FPIc. Furthermore, those response variables were negatively correlated with the colour parameters, WAC, WHC, and OAC, which were positively correlated with each other and positively affected by FWC.

### 3.2. Effect of Independent Variables on the Mechanical Properties of Meat Analogues

**Figure 2:**
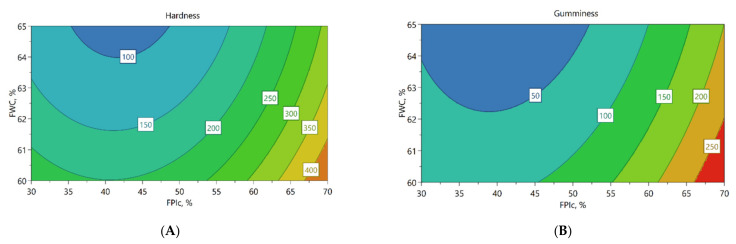

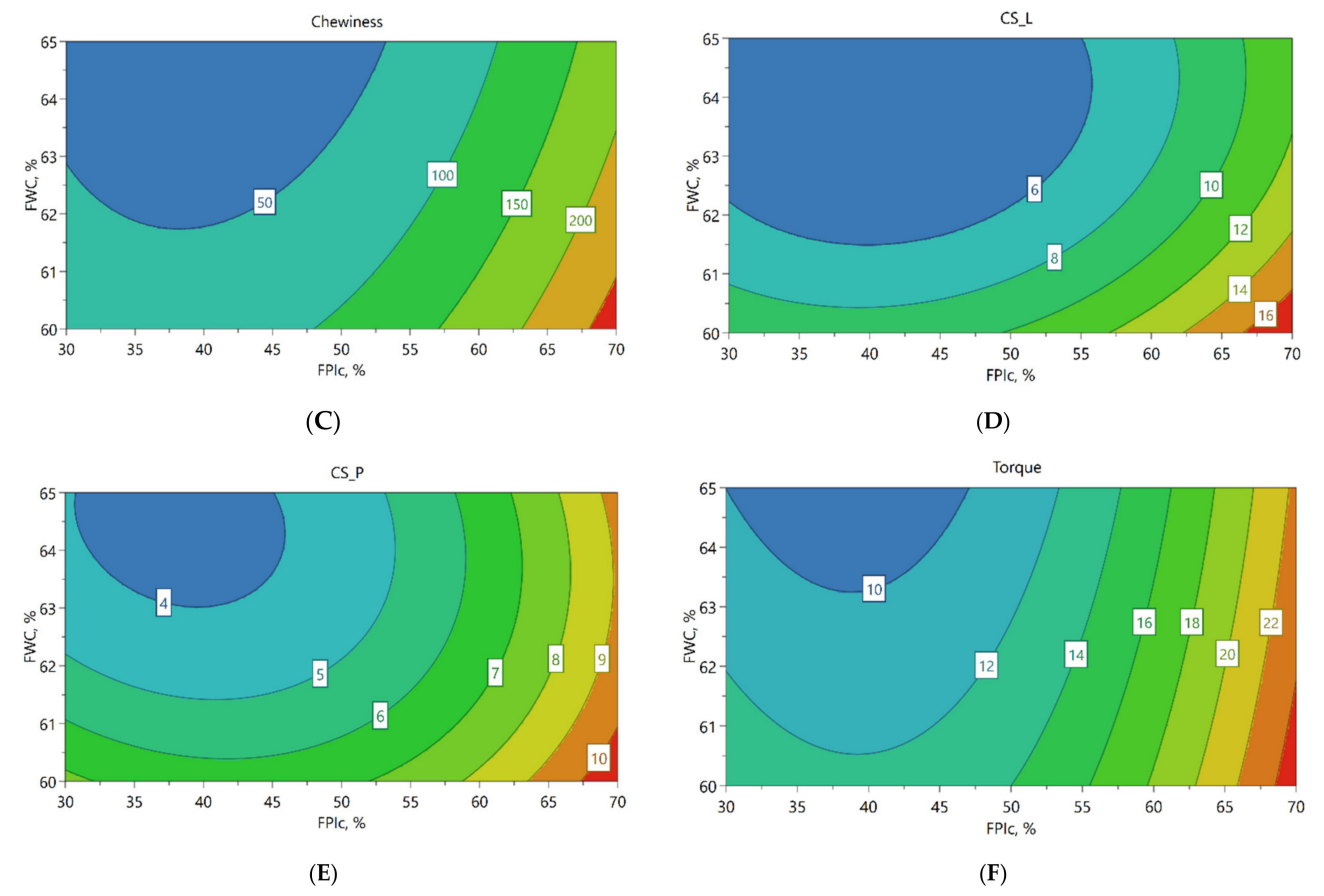
Contour plots from the MLR model showing the effect of feed water content (FWC) and faba bean protein isolate content (FPIc) on the hardness (**A**), gumminess (**B**), chewiness (**C**), cutting strength longitudinal (**D**), and cutting strength perpendicular (**E**) of the meat analogues and the torque (**F**) during extrusion, when the LT was 60 °C.

Texture profile analysis was conducted to measure the hardness, gumminess, chewiness, and springiness of the meat analogues, and the results can be found in Appendix B, Table A2. Higher FPIc led to harder, gummier, and chewier meat analogues, as shown in the contour plots (Figure 2A–C, respectively). By contrast, a higher FWC led to softer and less gummy and less chewy meat analogues. The contour plots also show that when FPIc increased from 50% to 70%, the increase in the values of the mechanical properties was clearer, and FWC seemed to have less effect than in lower FPIc.

The increase in the hardness, gumminess, and chewiness of the meat analogue caused by the increased FPIc could be explained by the increased protein content of the mixture (Table 1). As the protein content increases, more proteins are available for cross-linking, thus resulting in a firmer structure, as suggested by Zhang et al. [33]. Different results were obtained in a study by Ferawati et al. [13], in which the texture profile analysis showed a harder texture for the meat analogue prepared from FPC as compared to the meat analogue prepared from FPI. However, higher extrusion temperatures and lower water content were needed for the extrusion of FPC to obtain a constant flow and a fibrous texture, which affected the formation of a harder texture for the FPC-containing meat analogue. In the present study, a lower FWC was associated with higher values for the mechanical properties. Lin et al. [34] suggested that increased water content during extrusion results in higher water retention in meat analogues, thus decreasing the values of their mechanical properties. A higher amount of feed water during extrusion also leads to the lower viscosity and temperature of the melt, resulting in less severe extrusion conditions that decrease protein denaturation and texturisation [34,35].

Cutting strengths of the meat analogues were analysed from the perpendicular and longitudinal directions, and the results can be found in Appendix B, Table A2. Increased FPIc led to higher cutting strengths, whereas the effect of FWC was the opposite. Increasing the protein content of the mixture possibly resulted in more cross-linking between the proteins, thus increasing the cutting strength. Concerning the effect of FWC, similar results have been obtained in other studies, in which the effect of increased water content led to a softer structure and decreased cutting strength, possibly due to less cross-linking of the proteins [35,36]. Furthermore, FPIc seemed to have a stronger effect on cutting strengths when FPIc increased from 50% to 70%, and at these levels, the effect of FWC was lower (Figure 2D,E).

The differences between longitudinal and perpendicular cutting forces can be used to indicate the orientation of the fibres and the degree of texturization, also known as anisotropy index [37,38]. When the values of these cutting forces are close to each other, it indicates a lack of anisotropic structure [39]. In the present study, the AI values for all the samples, except sample 16, were below one, which means that the longitudinal cutting force was higher compared to the perpendicular cutting force (Appendix B, Table A2). This implies that the samples had more perpendicularly oriented fibres since more force was needed to cut across the fibres than between the fibres.

### 3.3. The Effect of Independent Variables on Torque and Pressure

Torque and pressure were recorded during the collection of each sample (Appendix B, Table A2), and the effects of different values of independent variables according to experimental design on torque and pressure were investigated. Increased FPIc led to higher torque, whereas increased FWC led to lower torque. Torque is affected by the viscosity of the melt, which, in turn, is affected by the feed water content and raw materials used in the extrusion [34,40]. Palanisamy et al. [35] observed that increased cross-linking and the higher viscosity of the melt created the need for more mechanical energy to rotate the screws, resulting in higher torque. The presence of soluble and insoluble polysaccharides in high moisture extrusion has also been shown to decrease melt viscosity and thus decrease torque [40,41]. In the present study, newly formed cross-links resulting from the increased FPIc possibly caused the increased viscosity of the melt, which could explain the rise in torque during the extrusion. The contour plot (Figure 2F) shows that the increase in torque was higher when the FPIc increased from 50% to 70% as compared to an increase from 30% to 50%. This indicates that at higher FPIc, the lowering effect of FWC on torque was less apparent. The PLS weight plot shows that torque during extrusion was positively correlated with mechanical properties and negatively correlated with the WAC and lightness of the samples.

The decrease in the viscosity of the melt has also been connected to a decrease in pressure during extrusion [38]. In the present study, according to the MLR model, increased FPIc and lower LT led to increased pressure. However, the Q^2^ for the model was poor.

### 3.4. Colour

**Figure 3:**
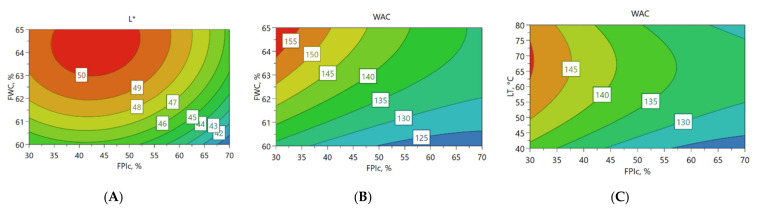
Contour plots from the MLR model showing the effect of feed water content (FWC) and faba bean protein isolate content (FPIc) on lightness (L*) (**A**) and on the water absorption capacity (WAC) (**B**) when the LT was 60 °C, and the effect of the long cooling die temperature (LT) and FPIc on the WAC (**C**) when FWC was 62.5%.

Colour parameters of L*, a*, and b* were measured from the samples, and the results can be found in Appendix C, Table A3. Increased FPIc led to darker coloured meat analogues, whereas FWC had the opposite effect. The contour plot (Figure 3A) illustrates a clearer decrease in lightness as the FPIc increased from 50% to 70% compared to an increase from 30% to 50%. The effect of increased feed water content on the increased lightness of meat analogues has also been observed by other authors [12,35,42]. A decreased amount of feed water leads to a higher viscosity of the melt, which increases the specific mechanical energy during extrusion. According to Fang et al. [43], increased values of specific mechanical energy during extrusion resulted in higher frictional heat, thus increasing the Maillard reaction that contributed to the dark colour formation. In the present study, the lower torque during the extrusion of raw materials with higher FPC content could also have led to lower frictional heat, thus resulting in lighter meat analogues. Furthermore, differences in amounts and oxidation reactions of phenolic compounds and proanthocyanidins in the raw materials may have led to colour differences between the samples with higher FPIc compared to the samples with lower FPIc [44]. The carbohydrate content could also have affected the colour of the extrudates. According to Chen et al. [40], the addition of starch increased the lightness of pea protein isolate meat analogues produced with the use of high moisture extrusion.

### 3.5. Water and Oil Absorption Capacity and Water Holding Capacity of the Meat Analogue

**Figure 4:**
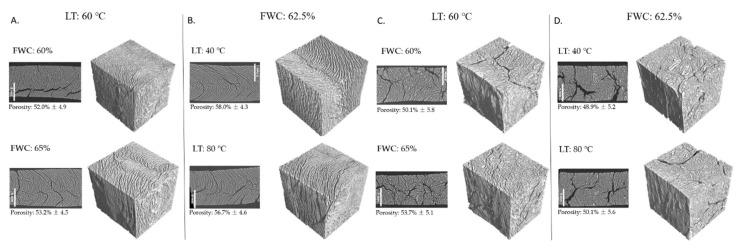
Micro tomography images and porosity values of the samples containing 30% (**A**,**B**) and 70% (**C**,**D**) faba bean protein isolate extruded at various long cooling die temperatures (LT) and feed water contents (FWC).

The results from WAC, OAC, and WHC of the samples can be found from Appendix C, Table A3. WAC increased with increasing FWC and decreased with increased FPIc. The lowest WAC was observed when FPIc was the highest and FWC was the lowest (Figure 3B). LT affected WAC, and the highest WAC was observed when LT was between 60 and 80 °C (Figure 3C). The PLS weight plot (Figure 1) shows a negative correlation between the mechanical properties and WAC, indicating that samples with harder structures had a decreased capacity to absorb water. WAC is defined as the amount of water retained in the sample after rehydration and is affected by the porosity of the meat analogue [27,45]. In the present study, microtomography imaging revealed that meat analogues with 70% FPIc had lower porosity compared to meat analogues with 30% FPIc (Figure 4). This could explain why higher protein content resulted in lower WAC, since it also led to a denser structure, preventing more water from being absorbed. According to Lin et al. [27], soy protein isolate extruded with the highest amount of feed water (70%) had the highest capacity to absorb water compared to samples extruded with lower amounts of feed water (60, 65%). They also observed that samples extruded with lower amounts feed water had no significant differences in their WAC, but it was noted that these samples were denser than the samples extruded at 70% feed water content and were thus able to absorb less water. Increased feed water content during the extrusion of soy protein isolate and green tea powder also led to increased WAC [46]. The effects of the independent variables on the water hydration capacity (WHC), according to the MLR (Table 2) and PLSR (Appendix A, Table A1) models, did not show any statistically significant effects. In the analysis of the WHC, the supernatant was quickly absorbed back into the samples after centrifugation. This could have influenced the observation of the supernatant, which might have affected the result.

### 3.6. Microstructure of the Samples

**Figure 5:**
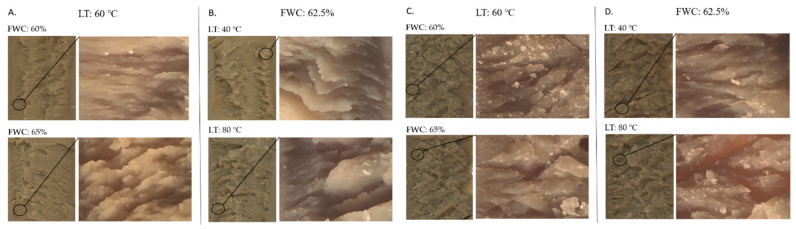
Pictures of samples taken with a digital camera and stereomicroscopy. Sample containing 30% faba bean protein isolate extruded at an LT of 60 °C (**A**), sample containing 30% faba bean protein isolate extruded at an FWC of 62.5% (**B**), samples containing 70% faba bean protein isolate extruded at an LT of 60 °C (**C**), and samples containing 70% faba bean protein isolate extruded at an FWC of 62.5% (**D**). LT = long cooling die temperature; FWC = feed water content.

Visual examination of the samples showed only minor differences in the fibrous structure of meat analogues extruded with different FWC when the FPIc and LT were kept constant (Figure 5A,C). According to Saldanha do Carmo et al. [12], increasing the feed water content in the extrusion of the faba bean protein concentrate led to a decreased anisotropic index, which was measured as the ratio between the perpendicular and longitudinal cutting forces. They also observed that samples extruded at higher temperatures and lower feed water contents had higher firmness but a lower anisotropic index, indicating a strong network formation but a lack of lengthwise fibres. As shown in the pictures in Figure 5C,D, samples containing 70% FPI seemed to have thicker layers and a less oriented fibrous structure than the samples containing 30% FPI (A and B). Microtomography imaging also revealed a more organised structure and more distinct fibrous structures in samples containing less protein (Figure 4). Furthermore, samples with 70% FPI were noted to be more brittle, and they fractured more easily compared to samples with 30% FPI. Kaleda et al. [47] also noticed that decreased protein content and increased carbohydrate content resulted in more fibrous structures of meat analogues produced from oat–pea protein blends in low moisture extrusion. According to Pietsch et al. [41], in the high moisture extrusion of the soy protein concentrate, non-protein components such as polysaccharides and their structural changes during extrusion influenced the rheological properties of the soy protein concentrate, which had an important role in the formation of anisotropic structures. Even though higher protein content in the raw material enables more cross-linking to occur, which would result in a tight protein network, it might not result in better fibre formation. Zhang et al. [48] studied the effect of transglutaminase in the high moisture extrusion of peanut protein and concluded that the addition of transglutaminase increased fibre formation up to a point, but excess addition resulted in a harder and less fibrous structure. In the present study, it was also evident from the visual examination that the samples had more perpendicularly than longitudinally oriented fibres. The results from the cutting strength analysis also support this, since cutting the sample required more force longitudinally.

**Figure 6:**
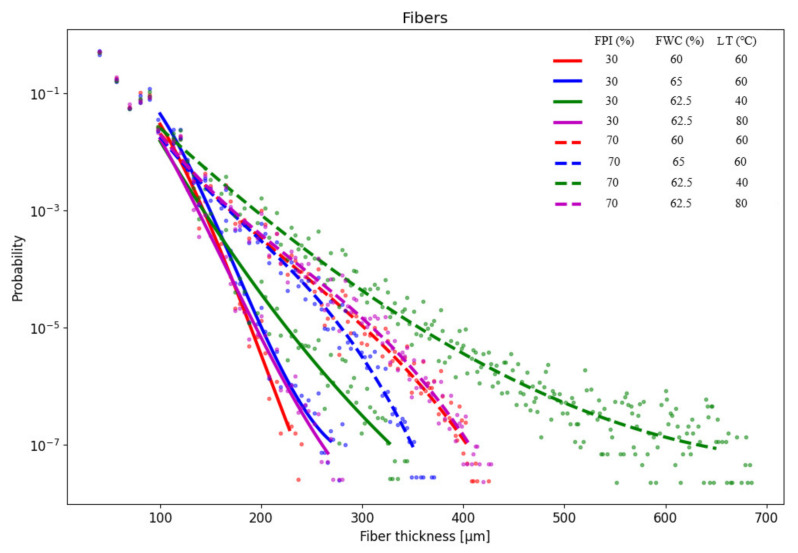
Results from the measurement of the fibre thickness of the meat analogues containing 30 and 70% FPI (faba bean protein isolate) at various feed water contents (FWC) and long cooling die temperatures (LT).

**Figure 7:**
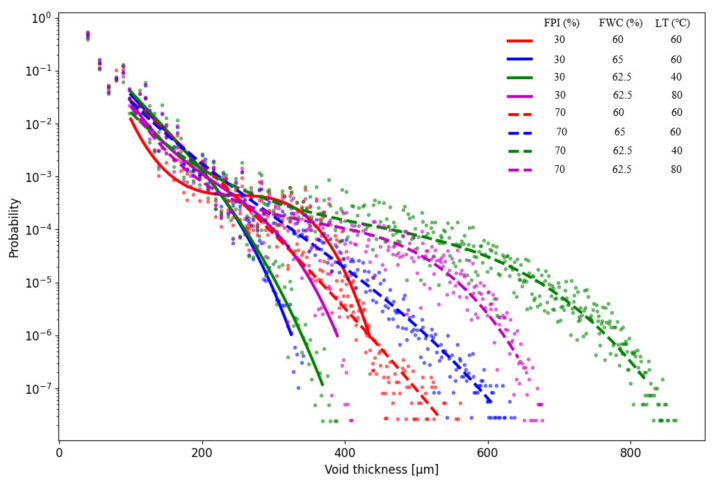
Results from the measurement of the void thickness of the meat analogues containing 30% and 70% FPI (faba bean protein isolate) at various feed water contents (FWC) and long cooling die temperatures (LT).

Micro tomography imaging was used to analyse the porosity, fibre thickness, and void thickness of the meat analogues. Meat analogues with 70% FPI had thicker fibres and larger voids compared to meat analogues with 30% FPI (Figure 6 and Figure 7). However, meat analogues with 30% FPI had higher porosity compared to meat analogues with 70% FPI (Figure 4). Results from fibre and void thickness measurements and from the visual examination of the microtomography imaging indicate that higher FPI content led to the formation of cracks between the protein structure. Microtomography imaging also revealed the formation of clusters in samples with 70% FPI, visible in a lighter grey colour in Figure 4. It has been suggested that carbohydrates in high moisture extrusion possibly enhance the phase separation into protein- and carbohydrate-rich phases, enabling the formation of anisotropic structures [40,47]. The clusters formed in samples with 70% FPI could be transversal protein aggregates due to the lack of phase separation caused by the incompatibility of proteins and carbohydrates [40]. Zhang et al. [33] also suggested that increasing the protein content of a plant protein mixture in high moisture extrusion resulted in excessive protein aggregation and led to the formation of gel-like structures instead of fibrous structures.

Vogelsang-O’Dwyer et al. [19] studied the functional properties of FPC produced by dry fractionation and of FPI produced by acid extraction and isoelectric precipitation. They found higher protein solubility and lower surface hydrophobicity for FPC, probably due to the presence of more native proteins than in FPI. They also stated that FPC had better functional properties related to its gelling ability and foaming capacity. In the present study, FPC was found to have higher WSI and lower WAI compared to FPI, which suggests that FPC contained more proteins in its native state. Whether the differences in the functionality of FPC and FPI affected fibre formation is only speculation and needs further investigation. Osen et al. [39] studied the high moisture extrusion of three different commercial pea protein isolates and the effect of their functional properties on the quality of meat analogues. According to their results, the differences in the functional properties of the pea protein isolates only had a minor effect on the fibre formation of the meat analogues. They speculated that the functional properties of the raw material are less significant at temperatures above of the proteins’ denaturation temperature. Likewise, Geerts et al. [49] concluded that the native state of plant proteins did not enhance the fibre formation in meat analogues produced with high-temperature shear cell. However, they noted that the higher water hydration capacity of the plant protein ingredient enhanced fibre formation. Furthermore, Pietsch et al. suggested [41] that the rheological properties of the raw material affected the formation of anisotropic structures by influencing the flow behaviour of the melt in the long cooling die. According to Wittek et al. [38], the viscosity ratio of the two phases in the melt could have affected the structure formation. Therefore, it could be assumed that the differences in the rheological properties of FPI and FPC rather than the differences in the functional properties caused by the state of the proteins possibly affected the structure formation. The differences in the rheological properties of FPI and FPC were suggested by the increase in torque when FPIc increased. As mentioned earlier, the higher the viscosity, the higher the torque.

## 4. Conclusions

Meat analogues with fibrous structures were achieved using all mixtures with different ratios of FPI and FPC. Higher FPIc led to higher torque during extrusion and to products with increased mechanical properties, a darker colour, and lower WAC. Higher FWC resulted in decreased values for the mechanical properties, a lighter colour, and higher WAC; however, this effect was less apparent at higher FPIc. The results from the microtomography imaging suggested that a higher FPI content led to the formation of a less oriented fibrous structure and to the formation of protein aggregates in the structure. The presence of soluble and insoluble polysaccharides in FPC could have positively influenced the fibrous structure formation by increasing the phase separation to protein- and carbohydrate-rich phases. Moreover, the rheological differences of the protein ingredients, as indicated by the difference in torque during extrusion, might have influenced the structure formation. This study provides new insights and criteria for the selection of plant protein ingredients to achieve optimised fibrous structures in meat analogues.

## Figures and Tables

**Table 1:** Chemical, physicochemical, and physical properties of the raw materials, including faba bean protein isolate (FPI) and faba bean protein concentrate (FPC) and their mixtures.

	Content g/100 g DM									
Raw Material	Protein	Dietary Fibre			Fat	Starch	WSI	WAI	Particle Size	
		Insoluble	Soluble	Total					D [2,3]	D [3,4]
FPI	80.1	9.7	1.0	10.7	4.8	0.3	11.6 ± 1.7	481 ± 7	32.7 ± 0.7	50.8 ± 0.3
FPC	55.2	17.6	2.3	19.9	4.9	7.3	56.3 ± 0.1	162 ± 1	6.8 ± 0.1	11.4 ± 0.1
Mixtures FPI:FPC										
70:30	72.6 *	12.1 *	1.4 *	13.5 *	4.8 *	2.4 *	24.6 ± 0.8	290 ± 14	15.6 ± 0.6	39.6 ± 0.2
50:50	67.6 *	13.7 *	1.7 *	15.4 *	4.8 *	3.8 *	31.2 ± 0.9	271 ± 30	10.2 ± 1.1	27.9 ± 4.5
30:70	62.7 *	15.3 *	1.9 *	17.2 *	4.9 *	5.2 *	41.9 ± 0.5	216 ± 1	9.3 ± 0.1	25.8 ± 0.4

WSI = water solubility index, WAI = water absorption index, D [2,3] = surface area moment mean, D [3,4] = volume moment mean. * Theoretical value based on the analysed chemical composition of the raw material.

**Table 2:** Split-plot Box–Behnken experimental design used, including independent variables of faba bean protein isolate content (FPIc), long cooling die temperature (LT), and feed water content (FWC).

Experiment Number	FPIc (% of Solids)	LT (°C)	FWC (%)
1	30	60	60
2	30	60	65
3	30	40	62.5
4	30	80	62.5
5	30	60	62.5
6	70	60	60
7	70	60	65
8	70	40	62.5
9	70	80	62.5
10	70	60	62.5
11	50	40	60
12	50	80	60
13	50	60	62.5
14	50	40	65
15	50	60	62.5
16	50	80	65
17	50	60	62.5

**Table 3:** Results of the multiple linear regression (MLR) analysis. Parameter estimates for the linear and quadratic effects and interactions of FPIc (content of faba bean protein isolate), FWC (feed water content), and LT (long cooling die temperature) on torque and pressure at the die, and on the hardness, springiness, gumminess, chewiness, cutting strength longitudinal (CS_L), cutting strength perpendicular (CS_P), water absorption capacity (WAC), oil absorption capacity (OAC), water hydration capacity (WHC), and the colour parameters L*, a*, and b* of the meat analogues.

	Torque	Pressure	Hardness ^b^	Springiness ^a^	Gumminess ^b^	Chewiness ^b^	CS_L	CS_P	WAC	OAC ^a^	WHC ^a^	L*	a* ^b^	b*
Constant	12.2 ***	4.73 ***	149.8 ***	0.90 ***	70.7 ***	63.2 ***	5.61 ***	4.81 ***	137.3 ***	19.6 ***	2.36 ***	49.2 ***	−0.23 *	9.9 ***
FPIc	6.00 ***	0.99 **	100.5 ***	0.02 **	89.0 ***	81.5 ***	3.55 ***	2.22 ***	−9.12 ***	−1.76 *	−0.06	−2.11 ***	−0.18 **	−0.03
FWC	−1.6 ***	−0.47	−60.2 ***	−0.01	−39.0 **	−35.9 **	−2.56 ***	−1.14 ***	9.35 ***	1.05	0.02	2.46 ***	−0.06	0.44 **
LT	−0.11	−0.8 **	−1.54	0.00	0.81	1.5	−0.37	0.14	4.11 *	−0.32	−0.02	−0.51	−0.13 **	−0.25
FPIc * FPIc	5.41 ***	0.38	115.6 **	−0.02	81.3 ***	71.7 ***	3.69 ***	2.22 ***	2.96	0.02	−0.04	−2.74 **	0.12	−0.23
FWC * FWC	0.24	−0.66	16.2	0.00	8.87	9.36	1.95 ***	0.86 *	−3.15	−2.32	−0.01	−1.47 *	0.07	−0.35
LT * LT	0.41	0.64	26.8	−0.01	19.27	15.7	0.55	0.69 *	−5.95 *	−1.59	0.00	−0.56	0.07	0.26
FPIc * FWC	0.22	−0.11	−7.91	0.00	−10.33	−10.8	−0.52	0.46	−3.41	−0.21	0.02	0.32	0.00	−0.19
FPIc * LT	−0.37	−0.83 *	31.3	0.02 *	17.41	18.4	−0.33	0.15	−1.03	1.72	0.01	0.20	0.02	0.28
FWC * LT	0.19	−0.02	−7.42	−0.01	−4.75	−4.9	−0.41	−0.04	−2.06	0.17	0.02	−0.07	−0.07	−0.29
R^2^	0.99	0.90	0.97	0.82	0.97	0.97	0.99	0.97	0.94	0.70	0.51	0.95	0.84	0.85
Q^2^	0.95	−0.08	0.83	−0.15	0.83	0.84	0.94	0.85	0.57	−2.57	−1.75	0.66	−0.81	−0.52

^a^ Regression is not significant at a *p* value of 0.05; ^b^ Lack of fit is significant at a *p* value of 0.05. * significant at *p* ≤ 0.05, ** significant at *p* ≤ 0.01, *** significant at *p* ≤ 0.001.

## Data Availability

The data presented in this study are available in article.

## Appendix A

**Table A1:** Results from the PLSR analysis showing the regression coefficients of the independent variables of faba bean protein isolate content (FPIc), feed water content (FWC), and temperature of the long cooling die (LT) on the response variables of torque, pressure, hardness, springiness, gumminess, chewiness, cutting strength longitudinal (CS_L), cutting strength perpendicular (CS_P), water absorption capacity (WAC), oil absorption capacity (OAC), water hydration capacity (WHC), and colour parameters of L*, a*, and b*, including the coefficient of correlation (R^2^), predictive square correlation coefficient (Q^2^), F-test, and root mean square error co-variant (RMSE_CV_).

Response Variables	Intercept	FPIc (%)	LT (°C)	FWC (%)	R^2^	Q^2^	F	RMSE_CV_
Torque	2.80	0.82	0.09	−0.26	0.75	0.70	6.86 **	2.99
Pressure	3.87	0.52	−0.12	−0.46	0.49	0.37	1.83	0.98
Hardness	2.17	0.73	−0.02	−0.39	0.68	0.62	4.95 *	65.32
Springiness	30.5	0.61	−0.04	−0.36	0.51	0.42	2.02	0.02
Gumminess	1.51	0.80	0.04	−0.33	0.74	0.70	6.87 **	46.81
Chewiness	1.47	0.80	0.04	−0.33	0.75	0.71	7.22 **	41.91
CS_L	2.29	0.72	−0.07	−0.46	0.73	0.67	6.35 **	2.15
CS_P	2.98	0.77	0.02	−0.34	0.71	0.69	6.79 **	1.23
WAC	11.65	−0.60	0.19	0.61	0.77	0.67	5.92 **	6.51
OAC	6.34	−0.51	−0.02	0.23	0.32	0.19	0.56	2.50
WHC	31.39	−0.61	−0.12	0.11	0.40	0.27	1.11	0.06
L*	15.95	−0.63	0.09	0.46	0.62	0.52	3.40 *	1.94
a*	−0.45	−0.71	−0.36	−0.24	0.69	0.49	2.78	0.15
b*	18.49	−0.18	0.13	0.30	0.14	−0.15	0.00	0.54
VIP		1.49	0.29	0.83				

* significant at *p* ≤ 0.05, ** significant at *p* ≤ 0.01.

## Appendix B

**Table A2:** Pressure and torque recorded during extrusion and results from the texture profile analysis; cutting strength longitudinal (CS_L), cutting strength perpendicular (CS_P), and the anisotropy index (AI) of the samples with different independent variables, including faba bean protein isolate content (FPIc), temperature of the long cooling die (LT), and feed water content (FWC).

Sample	FPIc (%)	LT (°C)	FWC (%)	Torque (Nm)	Pressure (bar)	Texture Profile Analysis				Cutting Strength		AI
						Hardness (N)	Springiness	Gumminess (N)	Chewiness (J)	CS_L (N)	CS_P (N)	
1	30	60	60	13.3 ± 0.6	4.1 ± 0.3	220 ± 20	0.9 ± 0.0	93 ± 12	80 ± 9	10 ± 2	6.9 ± 0.6	0.69
2	30	60	65	10.7 ± 0.3	3.4 ± 0.2	140 ± 20	0.8 ± 0.1	53 ± 12	45 ± 14	5.8 ± 0.4	3.8 ± 0.5	0.66
3	30	40	62.5	11.7 ± 0.4	4.9 ± 0.3	223 ± 10	0.9 ± 0.0	89 ± 7	80 ± 5	6.4 ± 0.6	5.7± 0.1	0.89
4	30	80	62.5	12.1 ± 0.4	4.1 ± 0.1	163 ± 11	0.8 ± 0.0	65 ± 6	54 ± 5	6.2 ± 0.3	5.7 ± 0.7	0.92
5	30	60	62.5	11.3 ± 0.3	4.0 ± 0.1	168 ± 11	0.8 ± 0.1	72 ± 6	60 ± 8	5.4 ± 0.6	4.9 ± 0.5	0.91
6	70	60	60	24.6 ± 1.2	5.7 ± 1.1	450 ± 20	0.9 ± 0.0	300 ± 20	270 ± 20	18 ± 2	11 ± 3	0.61
7	70	60	65	22.9 ± 1.0	4.4 ± 0.5	340 ± 20	0.9 ± 0.0	220 ± 20	196 ± 15	11.1 ± 1.5	10 ± 2	0.85
8	70	40	62.5	24.7 ± 1.2	9.0 ± 1.0	370 ± 40	0.9 ± 0.0	250 ± 20	220 ± 20	13.9 ± 1.5	9.1 ± 1.2	0.66
9	70	80	62.5	23.6 ± 1.4	4.8 ± 0.7	440 ± 50	0.9 ± 0.0	300 ± 40	270 ± 30	12 ± 3	9.8 ± 1.4	0.79
10	70	60	62.5	23.0 ± 2.0	6.6 ± 1.0	310 ± 30	0.9 ± 0.0	198 ± 12	175 ± 11	14 ± 2	10.2 ± 1.4	0.71
11	50	40	60	15.1 ± 0.5	5.5 ± 0.6	260 ± 30	0.9 ± 0.0	140 ± 20	120 ± 20	10.6 ± 0.5	7.5 ± 1.3	0.71
12	50	80	60	14.6 ± 0.5	4.8 ± 0.1	260 ± 20	0.9 ± 0.0	143 ± 10	130 ± 8	10.8 ± 1.0	8 ± 2	0.72
13	50	60	62.5	12.6 ± 0.5	4.4 ± 0.1	158 ± 12	0.9 ± 0.0	76 ± 8	68 ± 7	5.4 ± 0.2	4.1 ± 0.2	0.76
14	50	40	65	10.5 ± 0.4	4.7 ± 0.2	126 ± 10	0.9 ± 0.1	55 ± 8	48 ± 6	6.5 ± 0.8	5.2 ± 0.9	0.8
15	50	60	62.5	12.1 ± 0.5	4.6 ± 0.1	160 ± 20	0.9 ± 0.0	81 ± 9	72 ± 8	5.1 ± 0.4	4.6 ± 0.2	0.89
16	50	80	65	10.8 ± 0.4	4.0 ± 0.1	103 ± 11	0.9 ± 0.1	39 ± 6	33 ± 6	5.1 ± 0.7	5.4 ± 0.9	1.05
17	50	60	62.5	12.0 ± 0.4	5.0 ± 0.2	155 ± 4.0	0.9 ± 0.0	73 ± 5	67 ± 5	5.7 ± 0.6	5.2 ± 0.7	0.91

± shows standard deviation.

## Appendix C

**Table A3:** Water absorption capacity (WAC), oil absorption capacity (OAC), water hydration capacity (WHC), and colour parameters L* (0 = black, 100 = white), a* (− = green, + = red), and b* (− = blue, + = yellow) of the samples with different independent variables, including faba bean protein isolate content (FPIc), temperature of the long cooling die (LT), and feed water content (FWC).

Sample	FPIc (%)	LT (°C)	FWC (%)	WAC	OAC	WHC	Colour		
							L*	a*	b*
1	30	60	60	134 ± 3	16.7 ± 1.5	2.3 ± 0.1	44.1 ± 1.4	0.3 ± 0.2	8.9 ± 0.4
2	30	60	65	155 ± 12	19.0 ± 2.0	2.4 ± 0.1	49.4 ± 1.3	0.07 ± 0.10	10.1 ± 0.7
3	30	40	62.5	140 ± 4	25.0 ± 5.0	2.4 ± 0.1	48.2 ± 1.0	0.25 ± 0.15	10.4 ± 0.5
4	30	80	62.5	150 ± 3	18.0 ± 2.0	2.4 ± 0.1	47.2 ± 1.4	−0.06 ± 0.13	9.0 ± 1.0
5	30	60	62.5	151 ± 1	20.8 ± 1.3	2.4 ± 0.0	49.9 ± 1.2	0.01 ± 0.18	9.85 ± 0.9
6	70	60	60	126 ± 2	16.1 ± 0.5	2.2 ± 0.0	39.7 ± 0.7	−0.2 ± 0.2	8.8 ± 0.4
7	70	60	65	134 ± 2	18.0 ± 2.0	2.3 ± 0.1	46.3 ± 0.8	−0.46 ± 0.06	9.3 ± 0.1
8	70	40	62.5	121 ± 2	15.0 ± 2.0	2.3 ± 0.0	44.1 ± 1.0	−0.06 ± 0.13	10.2 ± 0.4
9	70	80	62.5	127 ± 3	15.0 ± 2.0	2.3 ± 0.0	43.9 ± 0.7	−0.31 ± 0.14	9.9 ± 0.4
10	70	60	62.5	129 ± 2	17.7 ± 0.7	2.2 ± 0.0	43.7 ± 1.2	−0.2 ± 0.2	9.9 ± 0.4
11	50	40	60	110 ± 3	13.0 ± 4.0	2.4 ± 0.1	46.0 ± 1.5	−0.07 ± 0.09	9.2 ± 0.3
12	50	80	60	123 ± 10	16.0 ± 4.0	2.3 ± 0.0	44.7 ± 1.0	−0.19 ± 0.08	9.6 ± 0.4
13	50	60	62.5	133 ± 7	21.0 ± 6.0	2.2 ± 0.0	49.9 ± 0.6	−0.22 ± 0.09	9.8 ± 0.6
14	50	40	65	137 ± 6	15.2 ± 0.7	2.4 ± 0.0	50.0 ± 1.0	0.14 ± 0.08	10.7 ± 0.3
15	50	60	62.5	144 ± 4	19.0 ± 3.0	2.4 ± 0.0	48.0 ± 1.4	−0.25 ± 0.04	9.94 ± 0.3
16	50	80	65	142 ± 5	18.0 ± 3.0	2.3 ± 0.1	48.4 ± 0.9	−0.25 ± 0.06	10.0 ± 0.2
17	50	60	62.5	136 ± 5	20.0 ± 4.0	2.5 ± 0.0	49.4 ± 1.3	−0.23 ± 0.07	9.8 ± 0.3

± shows standard deviation.
